# Integrative dual ctDNA 5mC/5hmC methylomics and clonal reconstruction infer tumor transcription and resistance phenotypes in metastatic prostate cancer

**DOI:** 10.21203/rs.3.rs-8778762/v1

**Published:** 2026-02-05

**Authors:** Chennan Li, Anna Baj, Clara C. Y. Seo, Nicholas T. Terrigino, John R. Bright, S. Thomas Hennigan, Isaiah M. King, Scott Wilkinson, Tzu-Ting Huang, Shana Y. Trostel, William D. Figg, William L. Dahut, David Y. Takeda, Jung-Min Lee, Fatima Karzai, Adam G. Sowalsky

**Affiliations:** 1 Genitourinary Malignancies Branch, National Cancer Institute, Bethesda, MD, USA; 2 Women’s Malignancies Branch, National Cancer Institute, Bethesda, MD, USA

## Abstract

Liquid biopsies can detect actionable mutations and infer broad tumor states from genome-wide cfDNA measurements, but quantitative transcriptome-like phenotyping at single gene resolution still largely requires tissue. Here, we asked whether 6-base whole-genome sequencing that jointly quantifies 5-methylcytosine (5mC) and 5-hydroxymethylcytosine (5hmC) could infer gene expression directly from plasma. We applied this framework to plasma from patients enrolled in a phase 2 clinical trial of the PARP inhibitor olaparib plus the PD-L1 inhibitor durvalumab for metastatic castration-resistant prostate cancer. Inferred plasma transcriptomes distinguished adenocarcinoma vs. neuroendocrine phenotypes and identified a noncanonical WNT5A-associated signature linked to poor clinical response. Integrating longitudinal cfDNA methylomic profiles with phylogenetic reconstruction further revealed two resistance trajectories: one featuring high tumor heterogeneity with persistent AR signaling, and another marked by an AR-independent, stem-like program with metabolic reprogramming. These findings demonstrate that ctDNA can inform phenotype-driven tumor biology at gene-level resolution, integrating epigenetic modifications, inferred transcriptional programs, and clonal dynamics as a function of treatment response.

## INTRODUCTION

Oncologic use of liquid biopsy approaches has expanded across solid tumors as a noninvasive adjunct to tissue profiling: for confirmation, assessment of treatment response and minimal residual disease, when evaluable tumor is difficult to acquire, and for serial monitoring [1, 2]. These assays generally rely on DNA shed into the blood as circulating cell-free DNA (cfDNA), of which a fraction originates from tumor cells (circulating tumor DNA, ctDNA). Across whole-exome or whole-genome sequencing, cancer gene panels, and tumor-informed assays, most approaches infer actionability, treatment response, or recurrence through detection of somatic mutations and copy number alterations [3]. While tumor genotypes can reflect altered biology through pathway activation or tumor suppressor inactivation, genotype-only analyses can miss critical changes in phenotype that drive outcomes and resistance, including tumor plasticity and dependence on alternative growth programs, particularly when ctDNA fraction is low or variable [4–6]. These phenotypes are instead typically identified using tumor RNA gene expression profiling, which enables molecular subtyping and can reveal therapeutic vulnerabilities that are not apparent from DNA alterations alone [7, 8].

Recently, efforts have emerged to exploit plasma cfDNA to infer tumor-specific patterns of gene expression in the absence of tissue. For example, fragmentomics leverages the genomic location and periodicity of plasma DNA fragmentation together with fragment-end motifs as proxies for chromatin organization, enabling inference of tissue-of-origin and broad transcriptional programs [4, 5, 9, 10]. Alternatively, cfDNA methylation/hydroxymethylation profiling can provide tumor-specific epigenetic signals and, in some settings, identify gene-level methylation features associated with tumor subtypes [11–13]. However, both approaches tend to be more informative at higher ctDNA fractions and generally do not provide global, gene-wise quantification of transcription at a resolution comparable to RNA profiling [12, 14]. Moreover, subtle gene expression changes accompanying resistance or progression may not manifest as large-scale reprogramming captured by fragmentomics or as changes in locus-specific methylation markers. An ideal noninvasive liquid biopsy assay in this setting would both predict benefit and enable early detection of treatment resistance through serial monitoring.

Metastatic castration-resistant prostate cancer (mCRPC) ultimately progresses through ongoing tumor evolution that drives resistance to systemic therapies [15, 16]. The androgen receptor (AR) remains the primary targetable axis in most metastatic disease [17], yet progression reflects selection for genomic alterations and regulatory reprogramming that modify lineage state and mitogenic programs [18]. In addition, metastatic sampling is frequently constrained by bone-dominant disease, and the multifocality and heterogeneity of mCRPC limit the extent to which a single time point of tissue captures ongoing genomic and epigenomic adaptation [7, 19, 20].

In this study, we applied a novel 6-base sequencing approach to cfDNA that captures A, C, G and T as well as 5-hydroxymethylated cytosines (5hmC) and 5-methylated cytosines (5mC) [21]. Because gene-body 5hmC is enriched at actively transcribed genes, whereas promoter-proximal 5mC is typically depleted at active genes, we hypothesized that integrating ctDNA genomics with plasma cfDNA epigenetic signals could infer transcriptional activity at gene-level resolution directly, without supervised learning models [22–24], yielding a gene-by-sample matrix amenable to standard transcriptome analyses. Using plasma collected from patients enrolled in a phase 2 clinical trial of the poly(ADP-ribose) polymerase (PARP) inhibitor olaparib plus the programmed cell death ligand-1 (PD-L1) inhibitor durvalumab for metastatic castration-resistant prostate cancer, we show that inferred gene expression links outcome to a WNT5A-associated, noncanonical WNT phenotype. Combining longitudinal plasma profiling with subclonal reconstruction further identified resistance trajectories associated with either rapid progression or clonal adaptation. Together, these data support a scalable framework for response stratification and early resistance monitoring that enables deeper study of tumor biology to inform rational combination therapy strategies.

## RESULTS

### Association of baseline ctDNA genomics and treatment outcomes

**Figure 1.**
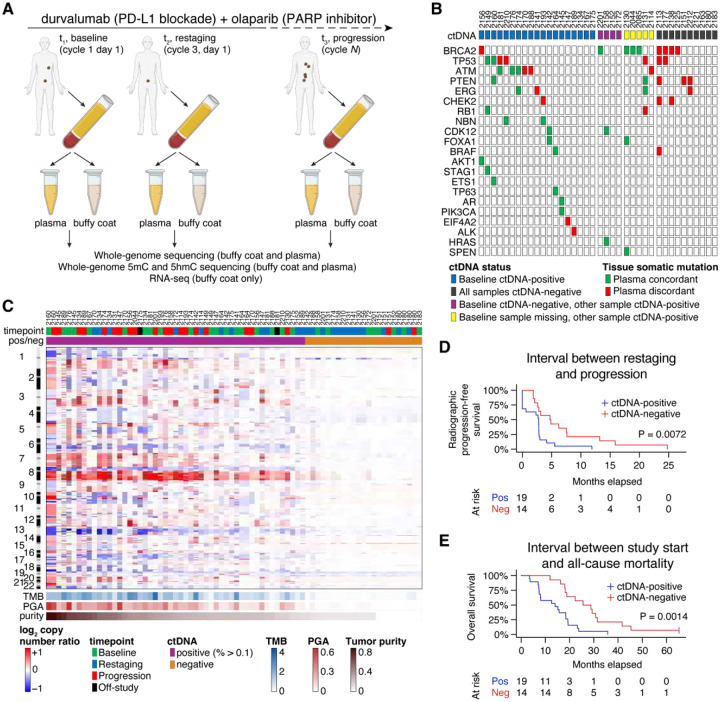
Circulating tumor DNA (ctDNA) detection in plasma samples. **(A)** Schematic of study design. Not all patients contributed all three timepoints due to rapid progression evident at the time of restaging and logistical/processing constraints. **(B)** Oncoprint depicting concordance of pathogenic somatic point mutations between time-matched tissue panel sequencing and plasma whole-genome sequencing. Concordance is also shown for cases lacking a baseline sample for which other timepoints are available. **(C)** Whole-genome estimates of SCNA in each plasma sample (determined with matched buffy coat control) ordered by tumor purity as measured by TitanCNA. SCNAs common in prostate cancer such as chromosome 8q gain and chromosome 13 loss are prominent in ctDNA-positive samples. **(D–E)** Kaplan-Meier estimates of radiographic progression-free survival **(D)** and overall survival **(E)** stratified by the detection or absence of ctDNA at baseline. P values are determined using log-rank test.

The overall goal of this study was to determine whether integration of orthogonal liquid biopsy-based approaches could assess the biology of metastatic prostate tumors in response to combined PARP and PD-L1 inhibition (olaparib and durvalumab, respectively) beyond identifying driver or targetable genomic alterations. As part of a phase 2 clinical trial of 60 patients (ClinicalTrials.gov NCT02484404), we collected plasma and buffy coat from 38 patients with metastatic castration-resistant prostate cancer who had previously progressed on AR-targeted therapies. We prespecified blood draws at baseline, on treatment at first restaging (cycle 3, day 1) and in patients who showed stable disease or at least partial responses at first or subsequent restaging, a third sample upon radiographic progression (Fig. 1A). Not all patients contributed all three timepoints due to rapid progression evident at the time of first restaging and logistical/processing constraints (including COVID-19-related disruptions). Baseline samples were obtained from 33 patients (Table 1); the complete three-timepoint series was obtained for 16 patients.

We performed deep whole-genome sequencing of plasma cfDNA (median coverage 140 ×) with paired buffy coat genomic DNA (gDNA, median coverage 153 ×). To quantify circulating tumor DNA (ctDNA) abundance in plasma cfDNA, we used titanCNA [25], an algorithm that relies on allelic copy number modeling. We further manually curated each sample using both tumor ploidy models and the presence of cancer-specific mutations. Overall, patients with a greater fraction of ctDNA in their plasma exhibited worse outcomes. ctDNA fraction at all timepoints was inversely associated with time to radiographic progression and overall survival (Supplementary Fig. 1A–F). Median ctDNA fraction at all timepoints was also greater in patients who exhibited progressive disease based on RECIST v1.1 criteria versus those with stable disease or partial responses (Supplementary Fig. 1G–I).

Interestingly, baseline ctDNA was only 55.7% concordant for confirming pathogenic alterations previously detected in on-study metastasis biopsies from targeted sequencing panels (Fig. 1B). However, we noted that discordance with tissue biopsies was due to ctDNA fractions close to zero, in which we failed to detect any valid mutations (Fig. 1B) or somatic copy number alterations (SCNAs, Fig. 1C) above background. We therefore defined samples with a ctDNA fraction threshold of < 0.1 as ctDNA-negative. This cutpoint meaningfully stratified our cohort: baseline ctDNA-positive patients demonstrated a shorter median time to radiographic progression (Fig. 1D, 2.8 vs. 4.8 months, P = 0.0072) and shorter median overall survival (Fig. 1E, 14.0 vs. 27.2 months, P = 0.0014). Similar survival advantages were also noted with lack of ctDNA detection in plasma acquired at restaging or progression (Supplementary Fig. 2A–D), in alignment with what has been reported for prior prostate cancer cohorts [26–28].

**Figure 2.**
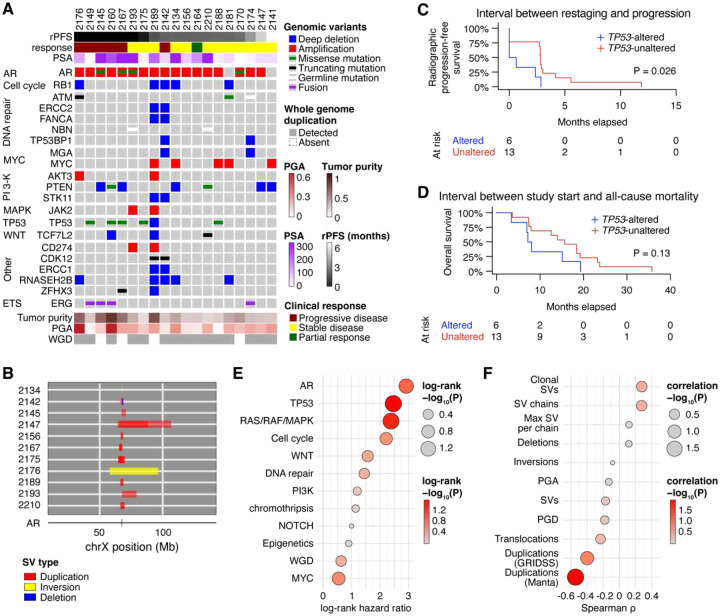
ctDNA carries limited genomic alterations informing treatment outcome. **(A)** Identification of genomic alterations in the baseline ctDNA-positive plasma. rPFS: radiographic progression-free survival; PSA: prostate-specific antigen measured at baseline; PGA: percent genome altered; WGD: whole-genome duplication. Genes that were mutated in at least two cases at baseline are shown. **(B)** Schematic of androgen receptor (AR) structural variants detected in baseline ctDNA. **(C–D)** Kaplan-Meier estimates of radiographic progression-free survival **(C)** and overall survival **(D)** stratified by the detection or absence of somatic *TP53* alterations at baseline. P values are determined using log-rank test. **(E–F)** Aggregation of gene-level somatic events into pathways and genome-wide observations and their association with rPFS, as determined by log-rank test **(E)** or Spearman correlation **(F)**. SVs: the abundance of structural variants; PGD: percent genome duplicated. Multiple comparison testing was not performed.

Given the wide range of ctDNA fraction (11.1–81.3%) in ctDNA-positive cases and the strong association between ctDNA-positive plasma at baseline and disease progression, we further explored the genomic landscape of baseline ctDNA. To identify any individual or groups of alterations associated with therapy outcomes, we curated each somatic copy number or single nucleotide variant using oncoKB and ClinVar. Given that all patients had previously progressed on AR-targeted therapies, it was expected that the most common genomic alteration was to AR (Fig. 2A), with 17 cases harboring *AR* amplifications, of which 11 were further resolved as structural variations focal to the gene or its upstream enhancer (Fig. 2B and Supplementary Fig. 3).

Strikingly, we observed very few cancer-related somatic alterations that were associated with clinical outcomes. Amongst all genes altered at baseline in at least two patients, only alterations to *TP53* detected in baseline ctDNA were associated with shorter median rPFS (Fig. 2C, 2.8 vs. 0.3 months, P = 0.026) and OS (Fig. 2D, 15.7 vs. 7.7 months, P = 0.13). As expected, germline variants impacting *BRCA2* were enriched in ctDNA-negative samples until progression (Supplementary Fig. 4A), consistent with its association with durable clinical responses to PARP inhibitors [29]. We detected the defective DNA homologous recombination mutational signature (SBS3) in 11 cases including all three harboring g*BRCA2*, suggesting a pre-existing homologous recombination-defective state not necessarily attributable to *gBRCA2* that resulted in a lower ctDNA fraction at the restaging timepoint, suggesting an initial favorable response. Subsequently, these cases that showed a greater ctDNA fraction upon progression (Supplementary Fig. 4B), and its detection was also associated with longer rPFS and OS (Supplementary Fig. 4C–D). By contrast, the defective DNA mismatch repair signature (SBS6), which might have been predicted to show favorable association with responses to PD-L1 inhibitors due to the potential for increased generation of neoantigens, was associated with shorter median rPFS (Supplementary Fig. 4E–F).

When we grouped all somatic mutations detected in baseline samples into annotated functional pathways [30] and patterns of genomic alteration, only alterations to the RAS/RAF/MAPK pathway and the number of duplications detected by Manta showed statistically-significant associations with shorter rPFS (Fig. 2E–F). The number of duplications was also significantly associated with OS, as well as alterations to the WNT pathway, the total number of SV chains, and the number of translocations detected (Supplementary Fig. 5A–B). Together, these findings suggest that baseline ctDNA genomics alone provides limited resolution of tumor phenotype and treatment resistance, thus promoting our further interest to interrogate this cohort using functional epigenomic profiling.

### Plasma cfDNA methylation recapitulates mCRPC functional and molecular subtypes

**Figure 3.**
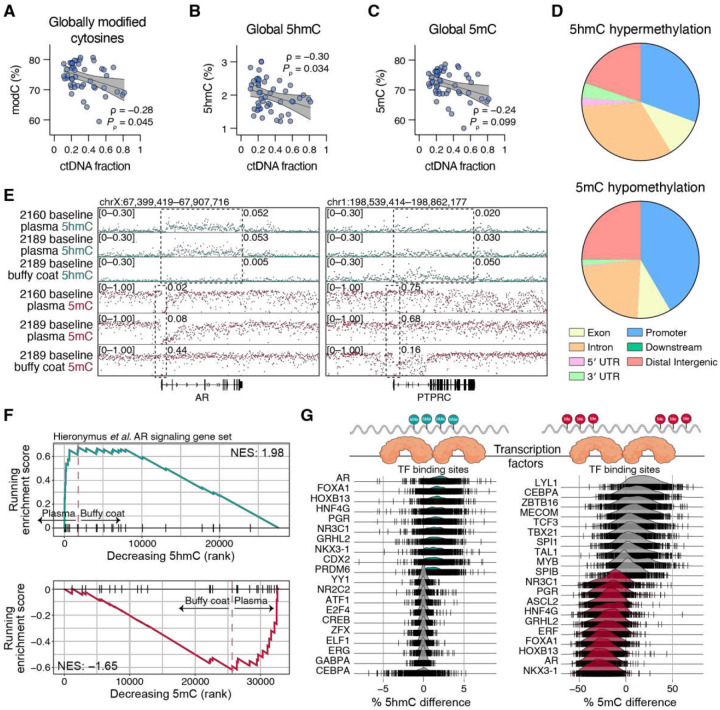
Plasma cfDNA methylation harbors prostate cancer-specific signals. **(A–C)** Correlation of the ctDNA fraction for each plotted against the total fraction of all modified cytosines **(A)**, hydroxymethylated cytosines **(B)** or methylated cytosines **(C)**. Nonparametric Spearman correlation analyses ρ values are shown with their respective P values. Lines and bands represent linear regression lines and 95% confidence intervals, respectively. **(D)** Global distribution of 5hmC hypermethylation (top) and 5mC hypomethylation (bottom) across genomic features. **(E)** 5hmC and 5mC profiles of representative plasma and buffy coat samples at the *AR* (left) and *PTPRC* (right) loci. Boxes demarcate the gene body (5hmC) and promoter (5mC) regions, annotated with normalized methylation fraction for those features. **(F)** Gene set enrichment analysis of six high-purity plasma cfDNA vs. buffy coat gDNA, ranked on the modified cytosine difference for the Hieronymus *et al*. AR signaling gene set. **(G)** Ridge plots illustrating the top and bottom 10 ranked candidate transcription factors (TFs) based on TF binding site differential hydroxymethylation (left) or methylation (right), plasma cfDNA vs. buffy coat gDNA.

To infer phenotype from ctDNA, we next turned to simultaneously detecting both 5-methylcytosine (5mC) and 5-hydroxymethylcytosine (5hmC) modifications using “6-base” DNA sequencing with the Biomodal duet-evoC platform [21]. Like somatic mutations carried by ctDNA, epigenetically modified tumor cell DNA is similarly protected in nucleosome-bound cfDNA. We sequenced all ctDNA-positive plasma-derived cfDNA samples plus nine buffy coat-derived DNA samples (median whole-genome coverage 23, Supplementary Table 1) using this approach to independently enumerate 5mC and 5hmC per CpG site. The mean proportion of modified cytosines (modC) at CpG sites across all samples was 75%, consistent with previous reports [31]. In cfDNA samples, the genome-wide proportion of modC, 5hmC and 5mC were inversely proportional to tumor purity (Fig. 3A–C, see Supplementary Table 1), in agreement with the canonical observation that global loss of DNA methylation accompanies cancer development [32]. Approximately 97% of all modC events were 5mC (Supplementary Table 1).

We collated the distribution of cfDNA 5mC and 5hmC events into genomic peaks, and as expected for mCRPC tumors, global depletion of 5mC (hypomethylation) was enriched at promoters and intergenic regions, whereas regions associated with increased 5hmC (hyperhydroxymethylation) were enriched at introns across the gene body (Fig. 3D) [33]. At the gene level, we confirmed promoter hypomethylation and gene body hyperhydroxymethylation for the prostate marker *AR* in cfDNA but not buffy coat samples, and vice-versa for *PTPRC* (CD45 gene) in buffy coat but not cfDNA samples (Fig. 3E). We further confirmed our ability to capture a prostate cancer phenotype from ctDNA methylation by examining differentially methylated genes in the Hieronymus *et al*. [34] AR signature using gene-set enrichment analysis (GSEA). Comparing high-tumor purity plasma vs. buffy coat samples, gene body 5hmC was enriched (NES = 1.98) and 5mC promoter methylation was depleted (NES = −1.65) in AR signature genes (Fig. 3F). This comparison similarly suggested depletion of gene body 5hmC enrichment (NES = 0.68) and enrichment of 5mC promoter methylation (NES = 1.74) against a panel of hematopoietic lineage genes (Supplementary Fig. 6A), consistent with the cellular content of buffy coat.

Differential DNA methylation patterns accompany histone modifications and chromatin accessibility, ultimately impacting systematic transcription factor (TF) binding [35]. Indeed, comparing the differentially hyperhydroxymethylated and hypomethylated regions of high-tumor purity plasma vs. buffy coat identified known TF binding sites (TFBSs) for prostate lineage factors, including FOXA1, NKX 3.1 and HOXB13, in plasma cfDNA (Fig. 3G). Conversely, differential hypohydroxymethylation and hypermethylation of TFBSs accurately inferred the activity of blood lineage TFs (such as CEBPA and LYL1) from the buffy coat DNA. Comparing the same high-tumor purity plasma against induced pluripotent stem cells (iPSCs) demonstrated enrichment for similar prostate lineage factors in plasma but enrichment for stemness factors (e.g. SOX2 and NANOG) in iPSCs (Supplementary Fig. 6B). Together, these data demonstrate the accuracy of using 5hmC or 5mC signals for identifying prostate cancer as a major source of cfDNA in patient plasma.

**Figure 4.**
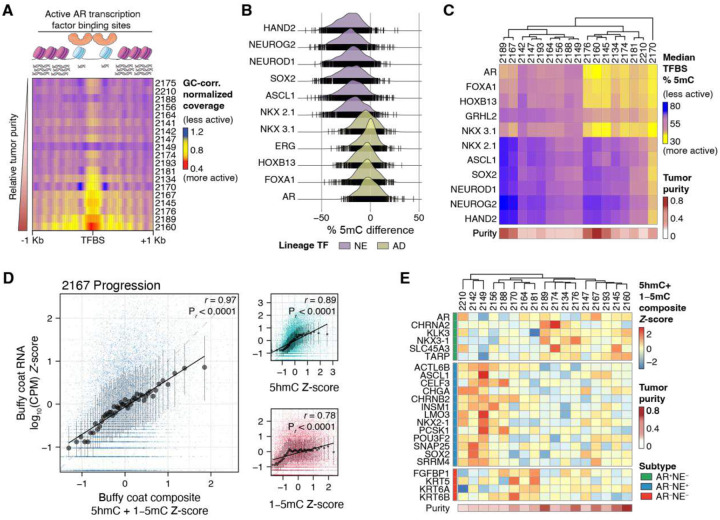
ctDNA-based tumor subtyping. **(A)** Per-sample depletion of sequence reads at 1000 AR binding sites, ordered by sample purity. Decreased central read coverage is associated with increased AR activity. **(B)** Ridge plot illustrating differential promoter hypomethylation comparing two cases with known mixed AD/NE pathology (2149 and 2170) vs. three cases with known AD pathology (2145, 2176 and 2189). **(C)** Heatmap depicting the predicted activity of lineage-defining TFs based on 5mC hypomethylation at known TFBSs. **(D)** Scatter plots of a representative buffy coat control sample depicting positive correlation between normalized expression from RNA-seq and a composite %5hmC gene body hypermethylation and inverse 5mC (1−%5mC) promoter hypomethylation Z-score. Pearson correlation analyses r values are shown with their respective P values. Points show the median *x* and *y* bin for all genes ranked by each Z-score metric and partitioned into 50 equal-sized quantile bins. Error bars depict the interquartile range. r and its and associated two-sided P value were computed using the binned medians. Black lines represent linear regressions. Individual genes are also shown. **(E)** Heatmap depicting gene-level expression estimates of prostate cancer subtype-defining genes based on the relative (Z-score) of the composite fractions of 5hmC gene body hypermethylation and 5mC promoter hypomethylation, per gene. A positive Z-score is predictive of greater gene expression.

We next asked whether we could harness ctDNA features to distinguish among cancer subtypes. Fragmentomics, a nucleosomal footprinting approach that relies on the deprotection of open chromatin from circulating nucleases, has been demonstrated to discriminate between metastatic adenocarcinoma (AR) and neuroendocrine (NE) prostate cancer subtypes via central coverage loss at AR binding sites in ctDNA [4, 5, 10]. Although fragmentomic analysis recapitulated predicted TF binding across 16 lineage-defining TFs in 8 high-purity baseline samples from our cohort (Supplementary Fig. 7), we observed that nucleosomal footprinting is highly dependent on sample purity (Fig. 4A), limiting its subtyping utility in our cohort. Therefore, we compared two samples with combined AD and NE pathology to three samples with known AD pathology and identified anticipated differential 5mC depletion at known NE and AD lineage-specific TFBSs (Fig. 4B). Extending this analysis to all 17 baseline samples, we noted that detection of AD or NE TFBS 5mC depletion was no longer dependent on tumor purity and more accurately reflected the metastatic phenotypic heterogeneity of prostate tumors to harbor both AD and NE subclones (Fig. 4C).

We performed 6-base-sequenced on nine buffy coat samples to serve as patient- and timepoint-specific controls for background correction. However, these controls also afforded us the opportunity to examine gene body 5hmC enrichment or 5mC promoter hypomethylation from samples with abundant RNA with which we could perform whole-transcriptome sequencing (RNA-seq). Across all nine buffy coat samples, gene-level epigenetic measurements tracked with RNA abundance, with the strongest concordance observed for a composite score combining both gene-body 5hmC and the inverse of promoter 5mC (calculated as 1−5mC). Quantile-binned medians showed proportional increases in RNA abundance with increasing composite epigenetic score, and the composite consistently outperformed either mark alone (Fig. 4D and Supplementary Fig. 8). For these nine samples we observed median correlation coefficients of 0.89 (range: 0.84–0.96) for 5hmC, 0.83 (range: 0.77–0.88) for 1−5mC, and 0.97 (range: 0.94–0.98) for the composite of 5hmC + 1−5mC, confirming that the observed fractions of 5hmC and 5mC modifications at the gene level are proportional to its relative expression measured by RNA-seq.

When applied to tumor-specific gene expression as measured in plasma, we also found that by subtracting an aggregate promoter 5mC background profile derived from the 9 buffy coat DNA from each of the plasma cfDNA samples, we observed more accurate ctDNA-specific methylation-based expression estimates as evidenced by tighter clustering of samples from the same individual by PCA (Supplementary Fig. 9). Thus, we derived a composite gene tumor-specific inference Z-score that included both timepoint-specific background- and purity-adjusted 5mC promoter hypomethylation and 5hmC gene body hypermethylation profiles, with 5mC also adjusted for somatic copy-number (see methods), and assembled these values into a gene-by-sample matrix used for all downstream transcriptome analyses. When we applied this gene inference Z-score against a panel of 23 lineage-defining genes for identifying transcriptional programs for AD, NE and double-negative (AD^−^/NE^−^, DN) tumors, we observed a clearer reciprocal pattern between AD, NE and DN genes (Fig. 4E) than with 5mC or 5hmC individually (Supplementary Fig. 10). Across a range of tumor purities, this Z-score recapitulated the expected subtype-specific gene programs that are usually defined by RNA-seq, supporting the premise that a composite 5mC and 5hmC score can be used to infer gene expression for further response-associated differential gene activity analyses.

### Comparison of plasma cfDNA methylation patterns identifies epigenetic and immunological programs associated with therapy outcomes

**Figure 5.**
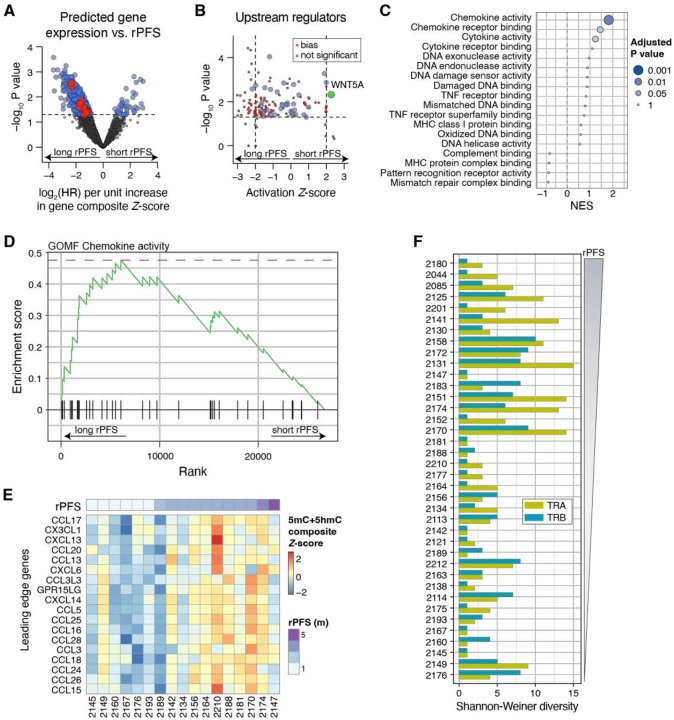
Baseline tumor intrinsic patterns of inferred gene expression associated with treatment response. **(A)** Volcano plot depicting the association of inferred composite gene expression with rPFS by Cox proportional hazards analysis. Red indicates genes whose inferred differential expression is consistent with WNT5A activation in patients with shorter rPFS. **(B)** Statistically significant gene Z-scores (P < 0.05) from Cox PH analysis depicted in **(A)** were used to infer upstream pathway regulators using the upstream regulator module of Ingenuity Pathway Analysis. A positive Z-score is associated with the risk of pathway activation in patients with shorter rPFS. **(C)** Gene set enrichment analysis using rPFS as a continuous variable assessed against a curated panel of gene ontology molecular function (GOMF) terms. A positive NES is associated with longer rPFS. **(D)** Mountain climber plot of the top GOMF term, Chemokine activity, associated with longer rPFS. **(E)** Heatmap of individual ctDNA inferred gene Z-scores for the leading edge genes of the analysis depicted in **(D)**. **(F)** Shannon-Weiner index for the T-cell receptor chain repertoire of the TRA and TRB gene clusters derived from MiXCR analysis of buffy coat whole-genome sequencing at baseline. Samples are ordered by rPFS with longer rPFS at the top.

To identify transcriptomic programs and pathways associated with treatment response, we next fit the composite gene inference Z-score from all baseline samples to a Cox proportional hazards (PH) model, such that genes with a positive log_2_ hazard ratio (HR) are associated with greater predicted expression in tumors with shorter rPFS, and a negative log_2_ HR is associated with genes with greater predicted expression in tumors with longer rPFS (Fig. 5A). This resulted in 1,082 genes statistically (P < 0.05) associated with rPFS that we applied to Ingenuity Pathway Upstream Regulator analysis (Fig. 5B) to identify potential tumor-intrinsic mediators of treatment resistance. The most activated upstream regulator (with lowest P value) in ctDNA from patients with shorter rPFS tumors was WNT5A (Z=2.27, P = 0.0046), a noncanonical Wnt signaling ligand (β-catenin independent) that has been implicated in both tumor immune evasion and alternative DNA damage sensing [36–38]. To disentangle these two potential mechanisms of resistance, we next performed gene set enrichment analysis on a curated set of Gene Ontology Molecular Function pathways related to inflammation and DNA repair (Fig. 5C–E), using rPFS as a continuous variable (see Methods). Enrichment for chemokine activity was most associated with longer rPFS (NES: 1.82, P_adj_ = 0.01), suggesting that more rapidly progressing tumors were, at baseline, already immunosuppressed and that sensitivity to immune checkpoint inhibition was due at least in part to tumor intrinsic inflammation.

To validate this phenotype, we similarly analyzed baseline WTS data (n=18) from the ovarian cancer cohort of this same clinical trial [39]. By Cox PH analysis, we observed significant enrichment for pathways associated with tumor-intrinsic interferon, T-cell receptor complex and co-stimulatory signatures amongst other growth repression signals associated with longer rPFS (Supplementary Fig. 11A–B). Unlike the prostate cohort, however, in ovarian cancer both defective DNA damage repair and intrinsic inflammation GO terms were associated with longer rPFS (Supplementary Fig. 11C–G), suggesting a consistent phenotype convergence towards increased intrinsic inflammation in responding tumors. We therefore applied the MiXCR algorithm to the WGS data from baseline buffy coat samples to assess T cell receptor diversity (Fig. 5F). Indeed, we observed significantly greater TRA locus diversity in patients with longer rPFS (Spearman ρ=0.46, P = 0.0033). In the prostate cancer cohort, this analysis revealed that immune suppression, rather than proficient homologous recombination, was likely to be a major driver of resistance in cases with more rapid radiographic progression amongst cases with detectable ctDNA.

### Longitudinal gene expression dynamics reveal divergent resistance phenotypes aligned with subclonal tumor architecture

**Figure 6.**
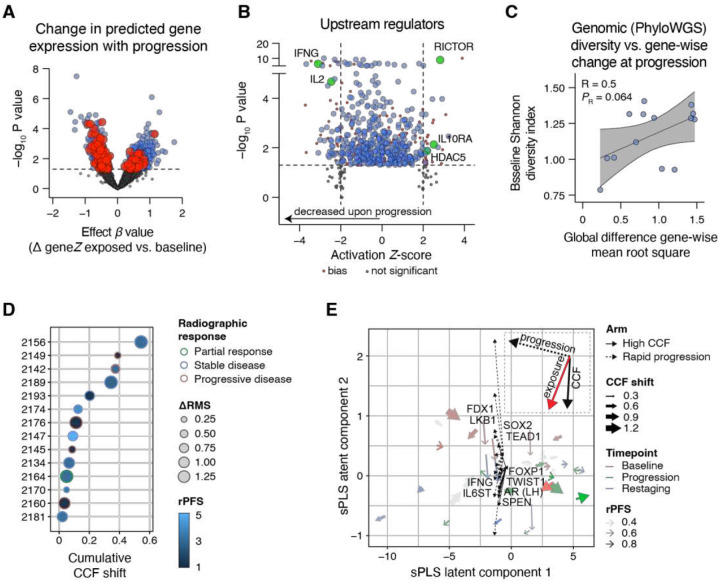
Integrated ctDNA transcriptional and genomic features define distinct evolutionary routes to treatment resistance. **(A)** Volcano plot depicting the paired gene-wise difference of inferred composite gene Z-score (ΔgeneZ) from upon treatment exposure using a linear mixed-effects model, with a random intercept for each case, testing the fixed effect of treatment exposure (restaging and/or progression vs. baseline). Red indicates genes whose inferred differential expression with exposure is associated with activation or inactivation of pathways highlighted in **(B)**. **(B)** Statistically significant gene Z-scores (P < 0.05) from eBayes analysis depicted in **(A)** were used to infer upstream pathway regulators using the upstream regulator module of Ingenuity Pathway Analysis. A positive Z-score is associated with activation in patients following treatment exposure. **(C)** Correlation of the Shannon diversity index (SDI, calculated using PhyloWGS) at baseline plotted against the global change in inferred gene expression, calculated as the difference in gene-wise mean root square (ΔRMS), for each pair of matched baseline and progression ctDNA samples with inferred gene expression (n = 14). Pearson correlation analysis *R* and P value is shown. Line and bands represent linear regression and 95% confidence interval, respectively. **(D)** Cumulative cancer cell fraction (CCF) shift for all subclones in each patient and depicted as a function of radiographic response, ΔRMS, and progression free survival (rPFS). **(E)** Multiblock sparse partial least squares (sPLS) latent space integrating ctDNA-derived upstream regulator scores and genomic features across time. Each point represents a sample; arrows indicate the direction of within-case change from baseline to restaging and/or progression. Black vectors depict IPA upstream regulators (|activation Z-score| > 1.5, P < 0.05) based on either a comparison of CCF shift (solid line) or rapid progression (dotted line), scored per sample by mean baseline-anchored ΔgeneZ as measured using empirical Bayes-moderated t-statistics. Inset: Bold vectors indicate the directionality and magnitude of the high CCF shift arm (solid line), rapid progression arm (dotted line), and the exposure axis (red line).

We next applied our estimates of gene expression in each ctDNA sample to ask if we could confirm a global change in phenotype upon exposure to combination durvalumab plus olaparib. Applying a linear mixed-effects model to baseline-anchored Z-score gene estimates for each sample vs. exposure timepoints (restaging and/or progression) we identified 1,539 genes (P < 0.05, Fig. 6A), for which Upstream Regulator analysis associated with both suppression of inflammatory regulators and activation of potential growth and epigenetic state change drivers (Fig. 6B). However, these globally observed phenotype changes may be due to numerous factors, including acquired or selected somatic mutations, changes in clonal abundance, and the duration of treatment exposure, which in turn is directly associated with intrinsic vs. adaptive resistance.

Nonetheless, we observed from our WGS somatic mutation analysis that the majority of potential genomic drivers in recurrent cancer genes (i.e. in at least two cases) did not change from baseline to restaging and/or progression (Supplementary Fig. 12). We thus also considered whether differences in the genomic composition of each tumor system impacted observed clinical responses. For example, three cases (2142, 2158 and 2189) harbored loss-of-function mutations to *cyclin-dependent kinase 12* (*CDK12*), and all displayed initial stable imaging responses (per RECIST) after 2 cycles followed by eventual disease progression (Supplementary Fig. 13A), which was accompanied by increases in ctDNA fraction (Supplementary Fig. 13B) and newly-emerged somatic mutations (see Supplementary Fig. 12). As expected, a genome-wide tandem duplication phenotype was prominent in the ctDNA-positive plasma samples from these patients (Supplementary Fig. 13C), arising from transcription and replication conflicts. By contrast, structural variants involving the *AR* gene were remarkably conserved across multiple timepoints from the same individual (see Supplementary Fig. 3) in patients spanning the entire clinical response spectrum, precluding AR as a major driver of treatment resistance in this setting.

Importantly, however, based on models of ctDNA-derived reconstructed tumor architectures, evidence of pre-existing subclones cooperating with driver (truncal) mutations may support a model of clonal selection in a subset of cases with shorter times to radiographic progression (Supplementary Fig. 14A). Although measures of baseline tumor diversity and outcome across the entire cohort were not significantly associated (Supplementary Fig. 14B–E), we observed a positive association between baseline tumor diversity and the global transcriptomic change from baseline to progression (as the root-mean-square (RMS) of gene-wise changes (ΔRMS) in baseline-anchored gene inference composite Z-scores, Fig. 6C). Despite noting significant ranges of subclonal cancer cell fractions (CCFs) shifts indicating a spectrum of adaptive to intrinsic resistance (Supplementary Fig. 15), we did not observe any clear association between within-case ΔRMS, radiographic response, rPFS, or CCF shift from baseline to progression (Fig. 6D). Given the wide variability in treatment-induced evolutionary pressures, we therefore explored non-mutually exclusive models of resistance that capture distinct axes of tumor behavior: evolutionary adaptation, quantified by CCF shift (“High CCF”, > 0.1) reflecting selection amongst competing subclones, and clinical response defined by rapid radiographic progression on therapy (rPFS < 2 months), indicative of intrinsic resistance. For each case with paired baseline and progression samples, we derived inferred signed gene-wise change vectors (ΔgeneZ) representing transcriptional shifts characteristic of each resistance phenotype. Upstream Regulator analyses of these signed gene lists identified different candidate regulatory programs associated with either clonal selection or rapid progression, confirming that treatment resistance reflects distinct evolutionary and phenotypic routes (see Source Data).

To integrate these pathways taking into account the genomic variability between (and within) cases we have already observed (see Fig. 2 and Supplementary Fig. 12), we constructed sample-level scores by projecting an unbiased candidate set of Ingenuity Upstream Regulator gene sets (|activation Z-score| > 1.5, P < 0.05) onto the baseline-anchored gene matrix, and combined these with per-sample genomic features (including somatic mutations, copy-number and structural variation summaries, WGD, and other curated genome-wide metrics). We then applied sparse multiblock partial least squares regression to embed samples into a shared low-dimensional space that summarizes coordinated variation over time. Cases showed variable trajectories from baseline towards progression, yielding a bifurcating phenotype model, wherein progression can align with either a greater CCF shift (adaptive) or a rapid progression (intrinsically resistant) arm (Fig. 6E).

Against a common phenotype of suppressed inflammation and increased growth signals that accompanied treatment exposure (see Fig. 6B), progression driven by clonal remodeling was aligned with both regulation of an androgen-dependent state (AR, FOXP1) and plasticity (TWIST1) and survival (SPEN). By contrast, rapid progression preferentially aligned with upstream regulators associated with stemness (SOX2) and metabolic reprogramming (LKB1, FDX1, TEAD1). Together, these data reveal that convergence to a state of clinically similar treatment resistance amongst genetically heterogeneous tumors is achieved via distinct phenotypes associated with either rapid progression or clonal complexity. Importantly, these findings argue against a single dominant resistance mechanism and instead define orthogonal phenotypic trajectories that can consider both tumoral clonal dynamics and clinical timing of radiographic progression.

## DISCUSSION

In this study, we combined gene-body 5hmC and promoter-proximal 5mC fractions from serial plasma samples to infer tumor gene expression at gene-level resolution. Because plasma tumor fraction varies widely in mCRPC [27], we implemented a straightforward deconvolution approach that normalized tumor-specific signals across samples and mitigated the impact of variable tumor purity, enabling meaningful comparisons between patients once ctDNA was detectable. This step leveraged time- and treatment-matched buffy coat as a background reference and incorporated copy-number correction for 5mC-derived measures. Z-scoring the composite 5hmC/5mC signal yielded a gene-level metric that correlated with RNA-seq and enabled downstream transcriptome-like analyses.

Treatment resistance in mCRPC is heterogeneous, arising through the cooperation of preexisting oncogenic drivers with therapy-induced selection and microenvironmental pressures [40]. Extending beyond genomic profiling of these drivers, assessing functional biology has been a longstanding goal of oncologic liquid biopsy analysis. This need is particularly acute in mCRPC, where tissue sampling for bone-dominant metastatic disease remains limited and where ctDNA fraction can be low or variable. Here, we demonstrate a scalable framework for noninvasive profiling with broad applicability to other cancers and clinical contexts where tissue is limited and serial phenotyping is needed.

Across tumor types, intratumoral diversity and treatment response underscore that longitudinal sampling must resolve both tumor state and clonal composition rather than relying on genotype alone, as evolutionary processes favoring multiple subclones can increase tumor fitness over time [41, 42]. In our cohort, recurrent mutations and copy-number alterations in canonical drivers were not sufficient to explain the range of clinical outcomes (see Fig. 2), prompting our efforts to derive functional biology robust to variable ctDNA fraction. Using the inferred gene-by-sample matrix, baseline analyses revealed lineage-state differences and identified outcome-linked transcriptional programs, including an immunosuppressive WNT5A-associated noncanonical WNT phenotype (see Fig. 5), illustrating how plasma methylomic gene inference can reveal clinically relevant tumor states without requiring metastatic tissue [36–38].

A distinctive component of this framework is that it supports longitudinal inference of tumor phenotype in the context of subclonal tumor genomics. By coupling plasma-derived epigenetic profiling to phylogenetic reconstruction from deep WGS, we linked changes in inferred transcriptional programs to shifts in clonal architecture over time, effectively integrating phenotype with evolutionary dynamics in the same samples. This combined analysis delineated at least two archetypal resistance behaviors (see Fig. 6E). In the first arm, resistance was dominated by subclonal diversity and clonal remodeling, with retention of androgen-axis activity and the rise of minor subclones under treatment selection. By contrast, the second arm was characterized by rapid progression of dominant baseline clones already harboring sufficient genomic alterations to evade therapy, consistent with an AR-agnostic, stem-like trajectory driven by SOX2 and the loss of lineage-defining *TP53* and *RB1*tumor suppressors [43–45]. Although these signatures and drivers should be interpreted as inferred phenotypic trajectories rather than validated causal mechanisms, they provide a clinically interpretable organizing framework for prioritizing orthogonal routes to treatment resistance.

Importantly, we evaluated these resistance trajectories in the context of a common therapeutic background in which tumors must evade both PARP inhibition and PD-L1 blockade. In our cohort, greater T cell receptor diversity in matched buffy coat at baseline tracked with longer rPFS (see Fig. 5F), consistent with cross-sectional studies across tumor types linking inflammatory phenotypes to response to checkpoint inhibition [46, 47]. We also observed treatment-associated suppression of interferon-γ (IFNG)- related programs at progression, supporting convergence on immune dysregulation upon radiographic progression [47]. Together, these findings suggest a practical paradigm in which features detectable in baseline plasma along with early on-treatment changes could be used to stratify patients, anticipate routes to resistance, and guide subsequent clinical decision-making.

This study has important limitations, including a modest sample size requiring cautious interpretations of findings and incomplete matched tissue validation. In addition, inferred transcriptional programs and upstream regulators will benefit from validation in independent cohorts with matched tumor multi-omics across broad ranges of tumor purity and gene expression. Nonetheless, the convergence of inferred ctDNA-based epigenetic phenotypes with genomic and clinical observations supports the robustness and translational potential of our longitudinal approach. By integrating plasma ctDNA 5mC/5hmC methylomics with phylogenetic reconstruction, we demonstrate an interpretable, transcriptome-like framework for longitudinal tumor phenotyping that is robust once ctDNA is detectable (ctDNA fraction ≥ 0.1). Together with standardized serial sampling and integration with additional imaging and clinical covariates, plasma-based phenotyping could complement existing cfDNA genotyping by enabling gene-level and phenotype-oriented monitoring to inform the optimization of rational therapy combinations.

## PATIENTS AND METHODS

### Study Approval

This multi-cohort phase 2 trial was approved by the Institutional Review Board of the Center for Cancer Research, NCI (Bethesda, MD; ClinicalTrials.gov Identifier: NCT02484404, first registered on June 25, 2015). This study was conducted in accordance with ethical principles that have their origin in the Declaration of Helsinki and are consistent with the International Council on Harmonization guidelines on Good Clinical Practice, all applicable laws and regulator elements, and all conditions required by a regulatory authority and/or institutional review board. Written informed consent was obtained from all patients prior to performing study-related procedures in accordance with federal and institutional guidelines.

### Biosample acquisition

Peripheral blood was drawn into Streck tubes as previously described [48] at study baseline (t_1_, cycle 1 day 1), at restaging (t_2_, cycle 3 day 1), and in patients that remained on study, upon radiographic progression (t_3_) (see Fig. 1A). Briefly, plasma was isolated using a two-spin protocol: first for 20 minutes at 300 × g at 22 °C, and the upper plasma layer was transferred for a second round of centrifugation at 5,000 × g at 22 °C for 10 minutes. The buffy coat was retained separately.

Cell-free DNA (cfDNA) was extracted from 0.25–3 mL plasma using the QIAamp Circulating Nucleic Acid Kit (Qiagen, cat. #55114) following the manufacturer’s protocol with minor modifications: buffer ACB was incubated with lysate for 10 minutes on ice and elution was performed twice with buffer AVE (25μL per elution) following a five-minute incubation at room temperature. gDNA was extracted from 10–100μL of buffy coat using the DNeasy Blood and Tissue Kit (Qiagen, cat. #69504) following the manufacturer’s protocol with two elutions of buffer AE (100μL per elution). RNA was extracted from 200μL of buffy coat using the NucleoSpin RNA Blood Kit (Macharay-Nagel, cat. #740200.50) following the manufacturer’s protocol.

### Library preparation and sequencing

Conventional whole-genome (4-base) sequencing libraries were prepared from plasma cfDNA and buffy coat-derived gDNA. Plasma cfDNA libraries were prepared using the KAPA HyperPrep Kit (Roche cat. #KR0961) from a variable input quantity of cfDNA, following the manufacturer’s protocol with minor modifications: the fragmenting and shearing steps were skipped, adaptor ligation was performed overnight at 4 °C, and the final number of PCR cycles was variable per-sample to generate 1μg of library. Buffy coat gDNA libraries were prepared using the KAPA HyperPlus Kit (Roche cat. #KR1145) from 250 ng of gDNA, following the manufacturer’s protocol with minor modifications: EDTA conditioning was performed based on a concentration of 0.5 mM EDTA, fragmentation was performed for 15 minutes to target a 350 bp fragment size, HyperPlus enzyme was used for end-repair and A-tailing, adaptor ligation was performed overnight at 4 °C, and 3 cycles of PCR were used to generate 1μg of final library. cfDNA and gDNA libraries were sequenced on NovaSeq S4 and NovaSeq XPlus 25B flowcells (Illumina) with 150 cycles paired-end.

Whole-transcriptome sequencing (RNA-seq) libraries were prepared from 0.1–1μg of buffy coat RNA using the NEBNext Ultra II Total RNA Library Kit with Globin and rRNA reduction (New England Biolabs), following the manufacturer’s protocol. Libraries were sequenced on a NovaSeq XPlus 25B flowcell with 100 cycles paired-end.

6-base whole-genome sequencing libraries were prepared from plasma cfDNA and buffy coat-derived gDNA using the duet multiomics solution evoC Kit (Biomodal) following the manufacturer’s protocol. Libraries were prepared using 5–15 ng of cfDNA (plasma) or 120–150 ng of gDNA (buffy coat). cfDNA and gDNA libraries were sequenced on NovaSeq XPlus 10B and 25B flowcells with 150 cycles paired-end.

### Variant calling analysis of 4-base whole-genome sequencing data

Sequencing adaptors were removed using trimmomatic (version 0.39) and reads were aligned to the human genome assembly (hg38 version) using the Burrows-Wheeler Aligner (BWA, version 0.7.17). Genome-mapped reads were sorted by coordinate, duplicates were identified using MarkDuplicatesSpark (GATK, version 4.2.6.1), NM, MD, UQ tags fixed using SetNmMdAndUqTags (Picard), and bases were recalibrated for quality using BQSRPipelineSpark (GATK). Reads from multiple flowcells were merged per sample, re-sorted by genomic coordinate, and marked for PCR duplicates again using MarkDuplicates. Median genome coverage was calculated using samtools depth (samtools, version 1.19). Median cfDNA/genomic DNA fragment size was calculated using CollectInsertSizeMetrics (Picard, version 3.10).

For germline variant detection, Haplotypecaller (GATK, version 4.2.6.1) was used with ERC set as GVCF and a reference SNP (dbsnp138.vcf) file used. Variants were assessed for quality using VariantRecalibrator (GATK) for SNPs and indels independently and then recalibrated using ApplyVQSR (GATK) with quality scores determined and ts-filter-level set to 99.5 for SNPs and 99.0 for indels. To identify potential functional impact of germline variants, ANNOVAR (version 2020-06-08) was used for variant annotation. Clinically pathogenic germline variants were identified using ClinVar.

For somatic singe nucleotide variant detection, Mutect2 (GATK, version 4.2.6.1) was used. A panel of normals (PON) was generated by calling variants in the buffy coat sample using Mutect2 in tumor-only mode with GenomicDBImport and CreateSomaticPanelOfNormals. Read contamination was calculated using GetPileupSummaries and CalculateContamination. Somatic variants were then detected in each plasma sample using Mutect2, while ruling out germline variants from matched buffy coat and the PON. Read orientation bias was assessed using LearnReadOrientationModel and variants were filtered using FilterMutectCalls with the read contamination and orientation bias previously calculated. Finally, somatic variants were annotated using ANNOVAR. All variants were curated for oncogenicity using OncoKB.

To identify post-hoc cross-sample contamination, germline single SNP site allelic frequency was determined in the matched buffy coats using GetPileupSummaries and used to detect potential contamination in each plasma sample using CalculateContamination with the argument match set to the matched buffy coat pile up summaries. Plasma samples with a contamination score greater than 0.1 were excluded from further analysis.

For somatic copy number variant (CNV) detection, a composite of GATK toolsets were used. Read depths and allelic counts were measured using CollectReadCounts and CollectAllelicCounts, respectively. Read depths from matched buffy coats were used to create a panel of normal using CreateReadCountPanelOfNormals. Background signals were then removed from tumor read depths using DenoiseReadCounts and the panel of normal generated. Normalized copy ratio was determined using ModelSegments which used the de-noised read depths and allelic ratios calculated previously, copy number states called using CallCopyRatioSegments, and modelled segments plotted using PlotModeledSegments. Genome-wide CNVs were visualized using Integrated Genome Viewer. Gistic 2.0 (version 2.0.23) was used to determine gene copy number. Oncogenic CNVs were called using oncokb-annotator (version 3.4) and manually inspected. Alternatively, titanCNA was used to model copy number variation independently. Tumor ploidy determined by titanCNA was manually curated based on allelic fraction pattern and the fact that a duplicated genome cannot turn into a non-duplicated genome over the treatment. Tumor purity was then determined based on the selected ploidy model using titanCNA. Whole-genome duplication status was called based on tumor ploidy.

For copy number-sensitive somatic structural variant (SV) detection, read depths and SNP allelic frequencies were first measured using COBALT (version 1.13) and AMBER (version 3.9), respectively. SVs were called using GRIDSS (version 2.13.2) and somatic SVs filtered using GRIPSS (version 2.3.2). PURPLE (version 3.1) was used to estimate tumor ploidy and copy number variations. SV clusters and potential driver events including gene fusions were then determined using LINX (version: 1.22). Quality-passing SVs with a tumor allele frequency (TAF, supporting fragments/total fragments) greater than 0.1 were identified and used to calculate the abundance of each type of SVs. Alternatively, MANTA (version 1.6.0) was used for somatic SV calling. SVs were functionally annotated using AnnotSV (version 3.3.6).

### Modeling of genomic instability

Chromothripsis events were called with ShatterSeek (version 1.1) using SVs determined by PURPLE and CNVs determined by titanCNA, and filtered using the criteria previously described [49].

### Phylogenetic modeling of tumor clonal structure

For phylogenetic reconstruction, multiple algorithms including phyloWGS, PhylogicNDT, and a cfDNA-sensitive toolset were used. For phyloWGS, somatic SNVs were determined by Mutect2 (VAF > 0.1, total depth > 70% of averaged depths at all sites, variants with < 90% strand bias) and CNVs determined were by titanCNA. The output of these tools was parsed using parse_cnvs.py (-f titan) and create_phylowgs_inputs.py (--vcf-type vardict). Phylogenetic analysis was performed using multievolve.py (--num-chains 40 –burnin-samples 1000 –mcmc-samples 2500), followed by write_results.py (--include-multiprimary --max-multiprimary 1).

For clonal modeling with PhylogicNDT, the clonal prevalence of each somatic variant identified by Mutect2 was first modeled using ABSOLUTE (version 1.0.6). PhylogicNDT (version 1.0) was then used to determine clonal architecture.

For the clonal inference algorithm based on Herbert et al. [4], sites with somatic variants present in any of the cfDNA samples per patient were pooled and used to call variants using the same Mutect2 pipeline described above in a joint calling mode. Somatic variants supported by at least eight counts and whose allelic frequency was greater than ten times of that from the buffy coat were collected. Heterozygous SNPs and log copy ratios determined by the GATK-CNV pipeline were also used to model clonality. Tumor ploidy and truncal copy number model were determined using copynum segment, and each tumor’s clonality and subclone truncal model were inferred using subclones cluster.

### Fragment-based transcription factor inference

For transcriptional activity inference using cfDNA fragmentation pattern (“fragmentomics”), Griffin [5] was used. GC ratio and sequence mappability were determined using griffin_GC_and_mappability_correction.snakefile and nucleosome occupancy was then quantified at each transcription factor binding site (TFBS) using griffin_nucleosome_profiling.snakefile. Selection of transcription factor binding sites were performed as described [50]. Nucleosome occupancy from thirty base pairs upstream to thirty base pairs downstream of each TFBS was averaged from all sites to calculate central coverage for each transcription factor.

### Sequence-based lymphocyte clonotype diversity analysis

Buffy coat WGS genome alignment files were subset to the genomic locations of *TRA/D*, *TRB*, *TRG*, *IGK*, *IGH*, *IGL* genes using samtools view (version 1.19) and converted to FASTQ using SamToFastq (Picard, version 3.1.0). MiXCR (version 4.6.0) was then used to evaluate CDR3 clonotype diversity with the following settings: exome-seq mode, and species=hsa.

### Processing and functional analysis of 6-base whole-genome sequencing data

To identify genomic regions with aberrant methylation, DNMTools (version 1.4.3) was used. Following the identification of methylation at CpG sites, dnmtools hmr was used to identify hypomethylated regions and dnmtools hypermr was used to identify hyperhydroxymethylated regions. CHIPseeker (version 1.44.0) annotatePeak was then used to annotate each identified region with the following setting: tssRegion −3000~3000, TxDb = TxDb.Hsapiens.UCSC.hg38.knownGene. The promoter was defined as one kilobase upstream of transcription start site.

To identify functional genomic elements including genes and TFBSs with aberrant methylation, methylKit (version 1.34.0) was used. CpG sites supported by a read depths of at least three per base were collected using methRead and compiled from each tiling window using regionCounts with a minimum base coverage of 10. To identify differentially methylated TFBSs, the same reference used for cfDNA fragmentomic analysis, GTRD was used. To identify differentially methylated genes, the 6-kb regions ranging from 1 kb upstream to 5 kb downstream of transcription start site were evaluated for 5mC patterns, whereas genebody was evaluated for 5hmC patterns. calculateDiffMeth was then used to determine differentially methylated regions.

To remove non-cancer signals from the mixture of observed 5hmC at each gene, we computed gene-wise background mean from matched buffy-coat controls $m_{g,t(s)}^{N}$ where t(s)∈{BL,TR} depending on whether sample s was from baseline (BL) or treated/exposed (TR, restaging and progression timepoints). Tumor-specific 5hmC $m_{g,S}^{T}$ is a derivation of a mixture model:

$$m_{g,s}^{T}=\frac{m_{g,s}^{\text{bulk}}-\left(1-p_{s}\right)\cdot m_{g,t(s)}^{N}}{p_{s}}$$

where $m_{g,s}^{\text{bulk}}$ is the observed bulk 5hmC fraction for gene g in sample s, and $p_{s}$ denotes the estimated tumor fraction (“purity”, ctDNA fraction) for sample s.

For 5mC, we similarly used a two-component mixture but accounted both for tumor purity and copy number. We define the effective copy number weights for tumor and normal contributions, where $CN_{g,S}^{\text{tumor}}$, is the tumor copy number at gene g in sample s (as measured by TitanCNA), and $CN^{N}$ is the normal copy number (2 for chromosomes 1–22; 1 for chromosomes X and Y):

$$CN_{g,s}^{T}=p_{s}\cdot CN_{g,s}^{\text{tumo}r}\;\text{and}\;CN_{s}^{N}=\left(1-p_{s}\right)\cdot 2$$

and derive tumor-specific 5mC $m_{g,s}^{T}$, as:

$$m_{g,s}^{T}=\frac{m_{g,s}^{\text{bulk}}\cdot \left(CN_{g,s}^{T}+CN_{s}^{N}\right)-m_{g,t(s)}^{N}\cdot CN_{s}^{N}}{CN_{g,s}^{T}}$$

To keep fractions proportional with inferred levels of gene expression, the $m_{g,s}^{T}$, was further transformed as the depleted fraction $d_{g,s}^{T}=1-m_{g,s}^{T}$.

We then performed gene-wise standardization across baseline 5mC and 5hmC as follows:

$$Z_{g,s}^{M}=\frac{d_{g,s}^{T}-\mu_{g}^{M}}{\sigma_{g}^{M}}\;\text{and}\;Z_{g,s}^{H}=\frac{m_{g,s}^{T}-\mu_{g}^{H}}{\sigma_{g}^{H}}$$

where $Z_{g,s}$ is the Z-score for either the 5mc (M) or 5hmC (H) modality, and $\mu_{g}$ and $\sigma_{g}$ are the mean and standard deviation across baseline samples for each gene within each modality. The composite per-gene Z-score was then computed as an equally weighted average of both 5mC and 5hmC Z-scores:

$$C_{g,s}=\frac{Z_{g,s}^{M}+Z_{g,s}^{H}}{2}$$

To derive gene expression estimates for the restaging and progression timepoints, we separately centered and scored each 5mC $\left(Z_{g,s}^{M}\right)$ and 5hmC $\left(Z_{g,s}^{H}\right)$ timepoint values:

$$Z_{g,s}^{M}=\frac{M_{g,s}-{\overset{‾}{M}}_{g}^{BL}}{\sigma \left(M_{g}^{BL}\right)}\;\text{and}\;Z_{g,s}^{H}=\frac{H_{g,s}-{\overset{‾}{H}}_{g}^{BL}}{\sigma \left(H_{g}^{BL}\right)}$$

where ${\overset{‾}{H}}_{g}^{BL}={\text{mean}}_{s\in BL}\left(H_{g,s}\right),\sigma \left(H_{g}^{BL}\right)={\text{sd}}_{s\in BL}\left(H_{g,s}\right),{\overset{‾}{M}}_{g}^{BL}={\text{mean}}_{s\in BL}\left(M_{g,s}\right)$, and $\sigma \left(M_{g}^{BL}\right)={\text{sd}}_{s\in BL}\left(M_{g,s}\right)$ for any sample s (baseline, restaging, progression). Similar to the baseline composite Z-score, each baseline-anchored timepoint composite Z-score was computed as:

$$Z_{g,s}^{\text{comp}}=\frac{Z_{g,s}^{M}+Z_{g,s}^{H}}{2}$$

To identify baseline transcriptional programs associated with outcome, we fit gene-wise Cox proportional hazards models using the composite Z-score from baseline samples and radiographic progression-free survival (rPFS). The hazard ratios and P values for genes with P < 0.05 (unadjusted) were used with Ingenuity Pathway Analysis.

To compare methylation differences between plasma and buffy coat samples, the AR-positive prostate cancer (ARPC) genes (derived from [51]) were evaluated for gene set enrichment analysis (GSEA, clusterProfiler) using the ranked gene list based on methylation difference (comparison of two phenotypes). To test the *a priori* hypothesis regarding tumor-intrinsic inflammation/immune signaling and DNA damage response/repair biology, baseline tumor inferred gene expression was also analyzed using preranked GSEA with progression-free survival (PFS) as a continuous phenotype. For each gene, we computed Spearman’s rank correlation between gene expression and PFS across patients, and genes were ranked by this correlation statistic. Gene ontology molecular function (GO:MF) Gene set names and definitions were obtained from mSigDB (human v2025.1.Hs, SQLite). Candidate gene sets related to tumor-intrinsic inflammatory/immune signaling and DNA damage response/repair were manually curated prior to analysis. Enrichment scores, normalized enrichment scores, and nominal P values were computed using the fgseaMultilevel implementation of GSEA, with multiple testing correction using the Benjamini-Hochberg method.

For longitudinal differential inferred expression, we fit gene-wise linear mixed-effects models using lme4 (lmer) with a case-specific random intercept: z_gene ~ Exposed + (1 | Case), Exposed (where Exposed was either the restaging or progression timepoint) vs. baseline, with Case as the patient identifier. The β (Exposed) coefficient is interpreted as the mean change in inferred baseline-anchored gene Z-score upon exposure relative to baseline. P values for the fixed effect were obtained using a Wald t-test with Satterthwaite degrees-of-freedom approximation without adjustment for multiple comparisons.

### Bulk RNA sequencing analysis

For validating 5mC and 5hmC gene estimates, RNA was extracted from buffy coats using the NucleoSpin RNA Blood kit (Macharay-Nagel). Strand-specific libraries were prepared using the NEBNext Globin and rRNA depletion library construction kit (New England Biolabs) and sequenced on NovaSeq XPlus 25B flowcells with 150 cycles paired-end. Following adaptor trimming using trimmomatic (version 0.39), sequence reads were mapped to the human genome assembly hg38 and gene counts were calculated using rsem-calculate-expression (STAR 2.7.11b, RSEM 1.3.3; --star --paired-end --estimate-rspd -p 30 --calc-ci --ci-memory 8192). To reduce the contribution of anucleated blood cells (that would not contribute DNA for methylation analysis), we excluded erythroid/hemoglobin and platelet/megakaryocyte-enriched transcripts prior to normalization and Z-scoring. The marker list included canonical globins (e.g., *HBA1, HBA2, HBB*) and additional erythroid structural/biogenesis genes (e.g., *ALAS2, SLC4A1, GYPA*), as well as platelet markers (e.g., *PPBP, PF4, ITGA2B, GP1BA*). Filtered RNA-seq counts were normalized to counts per million (CPM), log_10_-transformed, and converted to within-sample Z-scores for comparability to epigenetic measures.

For validating the functional impact of differential gene expression using an independent set of samples, FASTQ files from the ovarian cancer cohort of this study [52] were obtained. Adaptor trimming, mapping, and gene count normalization was performed as above.

### Trajectory analysis

For comparisons of treatment resistance trajectories, we first derived within-case transcriptional change vectors by computing a paired difference in baseline-anchored inferred expression for each gene, baseline vs. progression as the difference between composite Z-scores at each timepoint: $\Delta \;\text{gene}Z=\text{gene}Z_{\text{Progression}}-\text{gene}Z_{\text{Baseline}}$, restricted to cases with paired baseline and progression ctDNA methylation profiles. Cases were then stratified into two non-mutually exclusive resistance axes: clonal remodeling (where a high CCF shift was defined as a cumulative absolute CCF change of > 0.1 across all subclones for that tumor) and intrinsic resistance (where rapid was defined as radiographic progression occurring less than two months following the start of treatment).

For each axis, we tested for differential transcriptional change between groups using limma on the ΔgeneZ matrix, fitting a gene-wise linear model with group as the predictor and applying empirical Bayes moderation (eBayes) to obtain moderated t-statistics and two-sided P values for each comparison, without adjustment. The top 500 up-regulated and 500 down-regulated genes from each analysis were used with Ingenuity Pathway Analysis. The differential genes and pathways from the longitudinal linear mixed effects model were also included as the “exposure” axis.

To integrate these trajectory-associated programs with sample-level genomic alterations, we converted the top Ingenuity Upstream Regulators from each analysis (|activation Z| > 1.5 and overlap P < 0.05) into per-sample regulator scores by projecting each regulator’s target gene list onto the baseline-anchored geneZ matrix (scoring each regulator as the mean geneZ across its target genes present in the dataset). These regulator-score blocks (“exposure”, “rapid radiographic progression,” and “high CCF shift”) were combined with per-sample genomic features (including mutations, copy-number/SV summaries, and global genomic events) and analyzed using sparse multiblock partial least squares regression in mixOmics (block.spls, 2 components) [53]. Each ordinal timepoint (Baseline/Restaging/Progression) was specified as the response variable to produce a shared 2D latent space summarizing coordinated temporal variation across blocks.

We then summarized each trajectory arm by connecting the baseline centroid to the centroids of progression samples either high CCF shift or rapid radiographic progression cases. Each sample’s arm score was computed by projecting its 2D coordinates onto each arm direction, and case-level arm deltas were calculated as progression minus baseline arm score. The pathway vectors displayed on the 2D map correspond to upstream regulator features, oriented and scaled in the block.sPLS latent space to indicate the direction and relative contribution of each pathway or regulator to the high CCF shift and rapid radiographic progression arms.

### Statistics

Statistical analyses were performed with GraphPad Prism version 10, Microsoft Excel for Mac version 16, and R version 4.5.2 (R Core Team 2025). For comparisons across multiple groups, Kruskal-Wallis tests with Dunn’s multiple comparisons tests or Welch’s ANOVA with Dunnett’s multiple comparisons tests were used, as indicated. Continuous variables were compared between two groups using Welch’s t test, and categorical variables were compared using Fisher’s exact test. Associations were assessed using Spearman’s rank correlations or Pearson correlations. The outcomes of radiographic progression-free survival and overall survival were evaluated using Kaplan-Meier estimates with log-rank tests. Gene-level associations with radiographic progression-free survival were assessed using gene-wise Cox proportional hazards models. Longitudinal differential inferred expression was tested using gene-wise linear mixed-effects models with a case-specific random intercept, with Wald t tests and Satterthwaite degrees-of-freedom approximation. Differential transcriptional change between resistance groups was tested using two-sided empirical Bayes moderation. Gene set enrichment analyses were performed using preranked GSEA, with multiple testing correction by the Benjamini–Hochberg method. Pathway enrichment was filtered and sorted by P values and activation Z-scores.

## Supplementary Material

Supplementary Files

This is a list of supplementary files associated with this preprint. Click to download.

- supptext.docx

## Figures and Tables

**Table 1.** Patient characteristics of the cohort subset with baseline plasma collected.

Demographics (N=33)	Baseline ctDNA-positive (n=19)	Baseline ctDNA-negative (n=14)	P value
Median age [years (IQR)]	68 (60–73)	59 (53–65)	0.0082^a^
Race [% (N)]			
Black	10.5 (2)	21.4 (3)	
Asian	5.3 (1)	0 (0)	
White	78.9 (15)	78.6 (11)	
Unknown	5.3 (1)	0 (0)	
ctDNA detected [% (N)]			
After 2 cycles of therapy (SD/PR)	61.5 (8)	25.0 (3)	0.11^b^
Upon radiographic progression	100 (13)	33.3 (3)	0.0011^b^
Median on-study PSA [ng/ml (IQR)]	81.1 (30.2–373.9)	28.5 (10.9–84.3)	0.17^a^
Initial radiographic response [% (N)]			0.040^b^
Progressive disease	6	0	
Stable disease	12	11	
Partial response	1	3	
Median survival [months (IQR)]			
rPFS	2.8 (0–2.9)	4.8 (2.5–9.0)	0.0072^c^
Overall survival	14.0 (7.3–19.2)	27.2 (18.1–34.0)	0.0014^c^
Disease site [% (N)]			1^c^
Bone only	42.1 (8)	42.9 (6)	
Bone/soft tissue/viscera	57.9 (11)	57.1 (8)	

ctDNA: circulating tumor DNA. SD/PR: Stable disease or partial response. IQR: interquartile range. rPFS: radiographic progression-free survival.

a Welch’s t test.

b Fisher’s exact test.

c Log-rank test.

## Data Availability

The data underlying this article have been deposited in Database of Genotypes and Phenotypes (dbGaP) and Gene Expression Omnibus (GEO) at https://www.ncbi.nlm.nih.gov/gap/ and https://www.ncbi.nlm.nih.gov/geo/, respectively, and can be accessed with phs001813.v4.p1 (dbGaP) and GSE309976 (GEO).
